# MG53 inhibits cellular proliferation and tumor progression in colorectal carcinoma

**DOI:** 10.7150/ijbs.67869

**Published:** 2022-08-15

**Authors:** Pranav Gupta, Haichang Li, Guan-Nan Zhang, Anna Maria Barbuti, Yuqi Yang, Pei-hui Lin, Chuanxi Cai, Tao Tan, Jianjie Ma, Zhe-Sheng Chen

**Affiliations:** 1 Department of Pharmaceutical Sciences, College of Pharmacy and Health Sciences, St. John's University, Queens, NY 11439, USA.; 2 Department of Surgery, The Ohio State University, Columbus, OH 43210, USA.

**Keywords:** Cancer, Drug Resistance, ABCB1, rhMG53, Doxorubicin

## Abstract

Cancer is the second leading cause of mortality after cardiovascular diseases in the United States. Chemotherapy is widely used to treat cancers. Since the development of drug resistance is a major contributor towards the failure of chemotherapeutic regimens, efforts have been made to develop novel inhibitors that can combat drug resistance and sensitize cancer cells to chemotherapy. Here we investigated the anti-cancer effects of MG53, a TRIM-family protein known for its membrane repair functions. We found that rhMG53 reduced cellular proliferation of both parental and ABCB1 overexpressing colorectal carcinoma cells. Exogenous rhMG53 protein entered SW620 and SW620/AD300 cells without altering the expression of ABCB1 protein. In a mouse SW620/AD300 xenograft model, the combination of rhMG53 and doxorubicin treatment significantly inhibited tumor growth without any apparent weight loss or hematological toxicity in the animals. Our data show that MG53 has anti-proliferative function on colorectal carcinoma, regardless of their nature to drug-resistance. This is important as it supports the broader value for rhMG53 as a potential adjuvant therapeutic to treat cancers even when drug-resistance develops.

## Introduction

Cancer has long been known to be a heterogeneous disease that exhibits an uncontrollable growth of cells. 1, 2. Over the years, several efforts have been made to fight against cancer; the use of chemotherapeutic agents remains the most successful one. Each year the USFDA approves a number of agents for cancer treatment. Studies have shown that the extensive use of these agents leads to multidrug resistance (MDR), a condition in which tumor cells develop resistance to drugs that differ in their chemical composition and mechanism of action 3-7. MDR has known to be the major contributor towards the failure of chemotherapy and the mortality of cancer patients 8.

Out of the various factors causing MDR, the overexpression of a diverse class of drug efflux transporters, ATP-binding cassette (ABC) transporters, has been known to be the most aggressive one 3, 9, 10. These transporters are found on the plasma membrane of tumor cells and extrude chemotherapeutic drugs by utilizing energy derived from ATP hydrolysis 11-13. Amongst the 49 known human ABC transporters, ABCB1 is highly expressed in kidneys, intestine, adrenal glands, blood-brain barrier, and placenta 14. ABCB1 (commonly referred as P-glycoprotein (P-gp)) is the first member of the B family of ABC transporters 4, 15. Structural studies have reported that ABCB1 contains two nucleotide and two transmembrane binding domains 16. In these tissues, ABCB1 exerts protective function against cellular toxicants and xenobiotics. However, in the case of cancer cells, ABCB1 expression leads to a reduced cell concentration of the substrate chemotherapeutic drug, thus causing MDR 17, 18. Some of the most common substrate chemotherapeutic drugs of ABCB1 include paclitaxel, etoposide, vincristine, colchicine, and doxorubicin 19, 20. Many studies have been conducted to develop novel inhibitors as modulators of ABC transporter mediated MDR and as potential anti-cancer drugs 21, 22. These inhibitors sensitize the ABCB1 overexpressing cancer cells to the substrate chemotherapeutic drugs.

We previously identified MG53, a member of the tripartite motif/RING B-box Coiled Coil (TRIM/RBCC) family protein (TRIM72), to be an integral part of the proteins assisting in the repair of the cellular machinery 23, 24. MG53 mediates membrane repair by its redox-dependent oligomerization; thus, facilitating nucleation of intracellular vesicles to form repair patches at the site of injury 25. Proteomic analysis has shown that in species such as rat, mouse, human, and monkey, the amino acid sequence of MG53 remains preserved 26. Studies done in the past have shown that the membrane protective functions of MG53 can be translated into the treatment of clinical conditions such as acute lung injury, myocardial infarction, muscular dystrophy, wound healing and acute kidney injury 25, 27-30.

Chen and colleagues showed that knockout of TRIM72/MG53 through CRISPR-gene silencing led to aggressive lung tumor growth and metastasis in mice, raising the possibility that MG53 might possess tumor suppressor function in lung cancer 31-33. However, the anti-cancer effects of MG53 in colorectal cancer cells and tumor xenografts have not been studied. Colorectal cancer ranks at number three in terms of cancer-related deaths in the United States 2, 34. In 2020, approximately 148,000 people will be diagnosed with colorectal cancer with approximately 53,000 mortalities 34, 35. Thus, it is pertinent to develop novel therapeutic adjuvants for colorectal cancer. In this study, we show that MG53 inhibits proliferation of sensitive SW620 and doxorubicin-resistant and ABCB1 overexpressing SW620/AD300 cells and reduces tumor growth and progression in ABCB1 mouse xenograft models.

## Materials and Methods

### Chemicals

rhMG53 was purified from a *E. coli* fermentation system as described previously 25. Dulbecco's modified Eagle's Medium, fetal bovine serum (FBS), 0.25% trypsin, and penicillin/streptomycin were purchased from Hyclone (Pittsburgh, PA). The ABCB1 antibody was purchased from Thermo Fisher Scientific Inc. (Rockford, IL). The MTT reagent and DMSO were obtained from Sigma Chemical Co. (St. Louis, MO). Alexa FluorTM 647 protein labeling kit was purchased from Invitrogen (Waltham, MA).

### Cell lines and cell culture

The drug sensitive parental SW620 cell line and doxorubicin resistant SW620/AD300 cell line were as described previously 36. The present study also employed HCT-15 (from ATCC), S1, KB-3-1, and KB-C2 cells. These cell lines were cultured in DMEM media containing 10% FBS and 1% P/S at 37°C in a humidified atmosphere of 5% CO_2_.

### MTT assay

The sensitive and drug-resistant cells were trypsinized, seeded into 96 well plates and incubated for 24 h. The next day, cells were treated with 20 μl of rhMG53 at varying concentrations (0-100 μM). To assess the chemosensitizing effects of rhMG53, different concentrations of doxorubicin were added after pre-incubation with rhMG53 or verapamil for 2 h. After 68 h of treatment, MTT dye (4 mg/ml) was added and the plates were further incubated for 4 h. Subsequently, absorbance was determined at 570 nm by Opsys microplate reader (Dynex Technologies, Chantilly, VA) 36-38.

### Western blot analysis

Protein concentration was determined using the BCA assay from the treated and control cells. Twenty micrograms of protein was loaded and Western blotting was performed to measure the protein levels of ABCB1 using a previously described method and quantified using ImageJ 37. The ABCB1 (E1Y7S) rabbit mAb (catalog number 13978S) and β-Actin (13E5) rabbit mAb (catalog number 4970S) were purchased from Cell Signaling Technology (Danvers, MA) and used at 1:1000 dilutions.

### rhMG53 up-take and live cell confocal imaging

rhMG53 and bovine serum albumin (BSA) were labeled with Alexa FluorTM 647 protein labeling kit (InvitrogenTM, A20173) according to the manufacturer's instructions. SW620 and SW620/AD300 cells were cultured on glass bottom dishes (MatTek Inc., Ashland, MA). Alex647-rhMG53 or Alex647-BSA was added to the culture medium. Live cell images were taken by Nikon A1R confocal microscope and analyzed using ImageJ software.

### Experimental animals and methodology for generating MDR mouse tumor xenograft model

The athymic nude mice, purchased from Taconic Farms (Taconic Biosciences, NY), were housed at the animal facility of St. John's University. All experiments were reviewed and approved by the Institutional Animal Care and Use Committee (IACUC), as described previously 39, 40. The animal protocol approval number was 1899 and it was approved on 02/14/2017. The tumor xenograft model of SW620 and SW620/AD300 cells was generated as described previously 41. Animals were divided into four treatment groups: Group (a) vehicle (normal saline, q3d × 4, i.p.); (b) doxorubicin (2 mg/kg, q3d × 4, i.p.); (c) rhMG53 (2mg/kg, q3d × 4, i.v.); and (d) combination of rhMG53 and doxorubicin (2 mg/kg, q3d × 4, i.v., i.p.). The body weight of animals and tumor volume was accessed as described previously 42. The blood was taken via submandibular puncture on the last treatment day and white blood cells (WBC) and platelet counts were determined in all four groups. At the end, all the animals were euthanized, the tumor tissues were excised, and weighed.

### Statistical analysis

All the experiments were conducted in triplicates and ANOVA test followed with Tukey's post-hoc test to determine statistical significance at p < 0.05.

## Results

### Anti-proliferative effects of rhMG53 on parental and ABCB1 overexpressing cells

**Figure 1:**
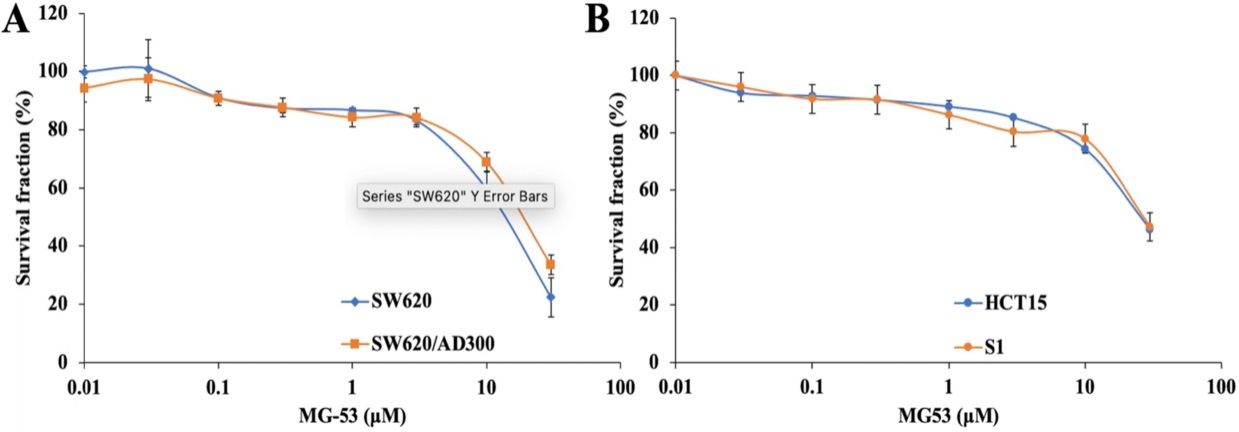
** Anti-proliferative effects of rhMG53 on parental and ABCB1-overexpressing cells.** Cytotoxicity assay was conducted to evaluate the anti-proliferative effects of rhMG53 on parental SW620 and ABCB1-overexpressing SW620/AD300 cells (A) and HCT15 and S1 cells (B). The data are the representation of mean ± SD for three independent experiments performed in triplicates.

In order to study the chemosensitizing role of rhMG53, its non-cytotoxic concentration was determined by MTT assay. Our results showed that over 85% of both drug sensitive and resistant cell lines were alive at the concentration of 3 μM rhMG53 (Figure 1), indicating that rhMG53 is non-toxic up to 3 μM (Figure 1A). In addition, rhMG53 did not exhibit significant cytotoxicity on two other human colon cancer cell lines, HCT-15 and S1 (Figure 1B). Based on these results, we selected 1 and 3 μM concentrations of rhMG53 to be investigated for their reversal activity in combination with a chemotherapeutic drug. At higher concentrations, rhMG53 reduced survival of both SW620 and SW620/AD300 cells.

### Anti-proliferative effect of rhMG53 does not involve changes in ABCB1 function

The drug sensitization effect of rhMG53 on ABCB1 substrate was investigated by performing the MTT assay. As shown in Table 1, SW620/AD300 cells were 315-fold resistant to doxorubicin as compared to the sensitive SW620 cells. Studies from our group have shown that SW620/AD300 also confers resistance to other ABCB1 substrate drugs such as paclitaxel, vincristine, and colchicine but not cisplatin 43-45. rhMG53 (up to 3 μM) did not significantly decrease the IC_50_ values of the ABCB1 substrate drug doxorubicin in the SW620/AD300 cells. These results suggested that rhMG53 did not sensitize the drug resistant SW620/AD300 cells to doxorubicin. Verapamil, a widely known ABCB1 inhibitor, was used as a positive control inhibitor of ABCB1, which did reduce the IC_50_ of doxorubicin from 56.78 ± 2.35 to 1.22 ± 0.21 (p < 0.05)**.**

Similar findings were also observed using KB-3-1 and ABCB1-overexpressing KB-C2 cells derived from HeLa, a cell line widely used for the study of MDR 46, 47. As shown in Supplemental Figure S1 and Table S1, rhMG53 can inhibit the growth of both KB-3-1 and KB-C2 cells without alteration of the IC_50_ of KB-C2 cells to doxorubicin treatment.

**Figure 2:**
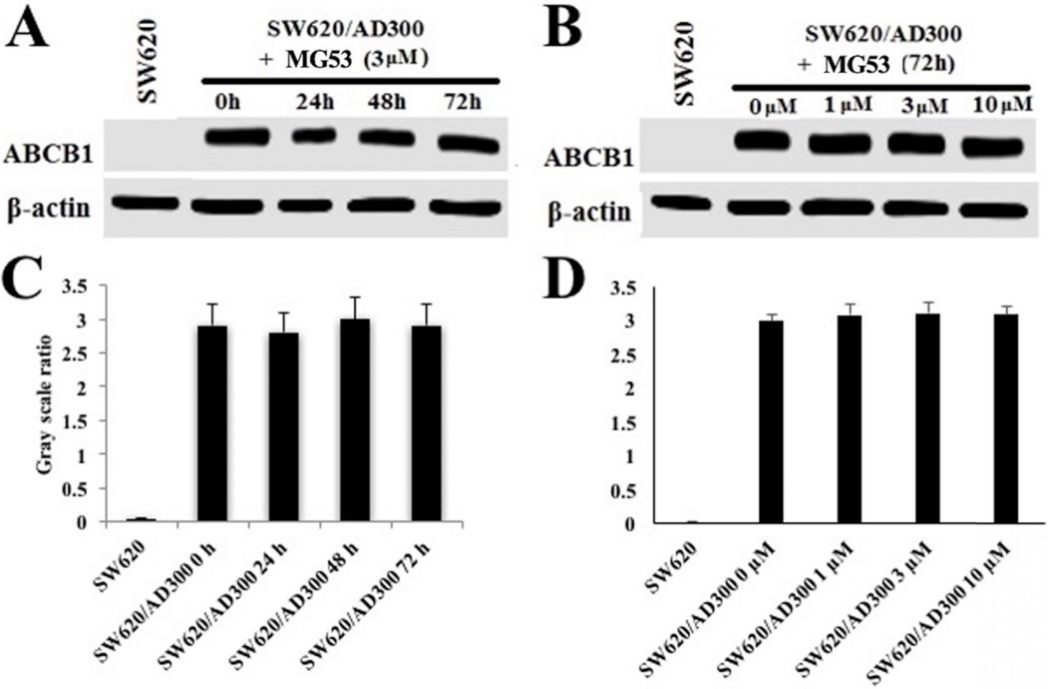
** Effect of rhMG53 on the expression of ABCB1.** SW620/AD300 cells were treated with rhMG53 at different time points (A) or different concentrations (B). Similar amounts of cell lysates were used, and Western blot analysis was conducted. The band intensity of ABCB1/β-actin was obtained with Image J and labeled as grayscale ratios (C, D). The differences presented are not statistically significant (p > 0.05).

To assess the effect of rhMG53 on the protein expression of ABCB1, Western blot analysis was performed. As shown in Figures 2A and 2C, treatment with rhMG53 (3 µM) did not significantly alter the expression of ABCB1 protein in SW620/AD300 cells following different times of incubation. Similarly, no changes in protein expression of ABCB1 were observed when SW620/AD300 cells were incubated with rhMG53 at indicated concentrations (1, 3, and 10 µM) for 72 h (Figure 2B and 2D). These results suggest that rhMG53 does not change the expression of ABCB1 when treated in a concentration and time-dependent manner.

### Uptake of rhMG53 in SW620 and SW620/AD300 cells

**Figure 3:**
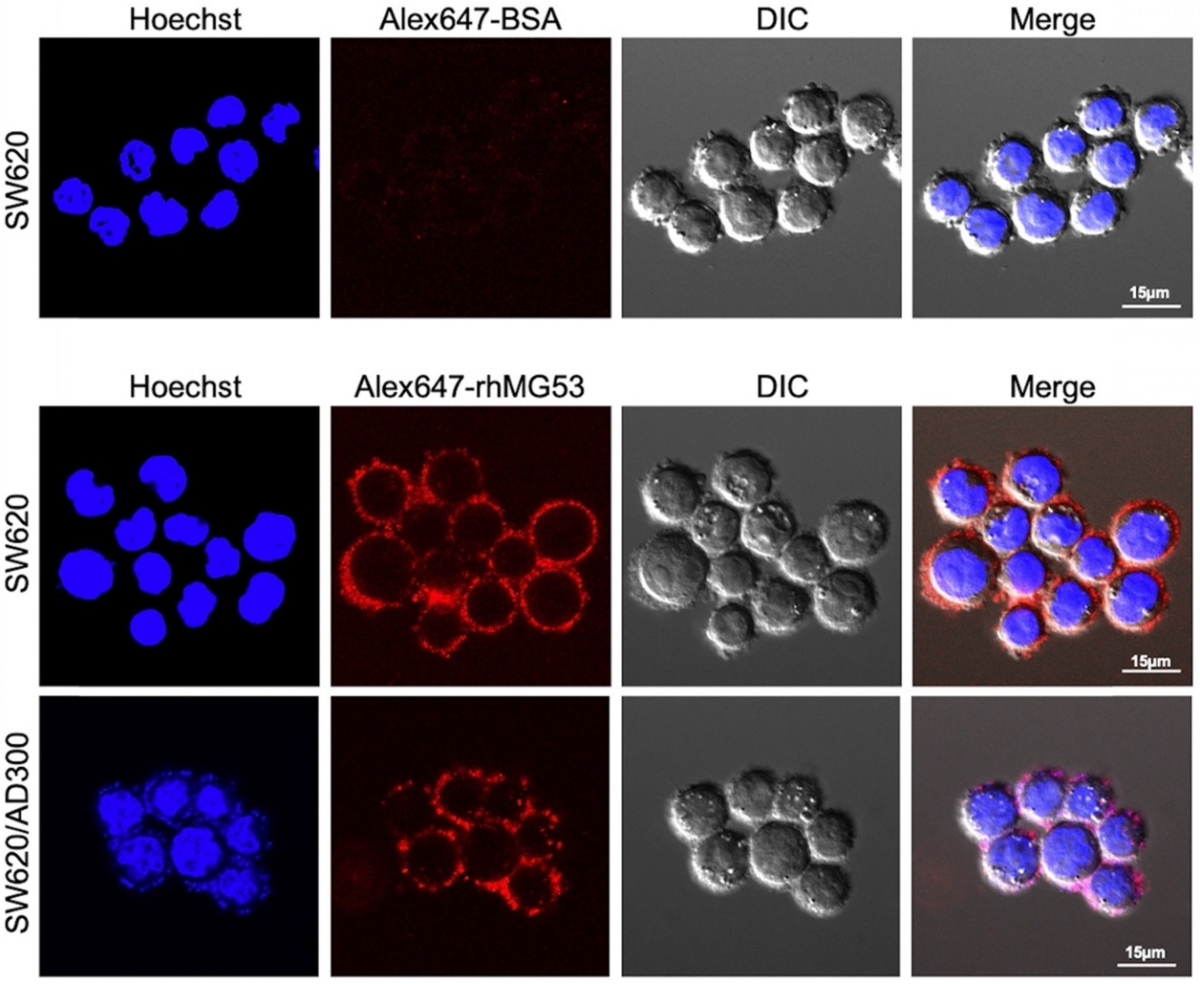
** rhMG53 up-take by colorectal adenocarcinoma cells.** Alexa647-rhMG53 or Alexa647-BSA was added to the culture medium of SW620 and SW620/AD300 cells. Confocal live cell images show that Alexa647-rhMG53 (red) localizes on the cell membrane and enters the cytosol after 2-hour incubation in both SW620 (middle) and SW620/AD300 (bottom) cells. As control, Alexa647-BSA shows no labeling of these cancer cells (top). The cell nuclei were stained with Hoechst 33342 (Blue).

To assess whether rhMG53 entered the SW620 and SW620/AD300 cells, the cells were incubated with Alexa FluorTM 647 labeled rhMG53, according to the manufacturer's instructions. Our results showed that Alexa647-rhMG53 (red) localizes on the cell membrane and enters the cytosol after 2-hour incubation in both SW620 (middle) and SW620/AD300 (bottom) cells (Figure 3). As control, Alexa647-BSA shows no labeling of these cancer cells (top).

### Effect of rhMG53 on the volume and weight of tumors in ABCB1-overexpressing tumor xenograft model

**Figure 4:**
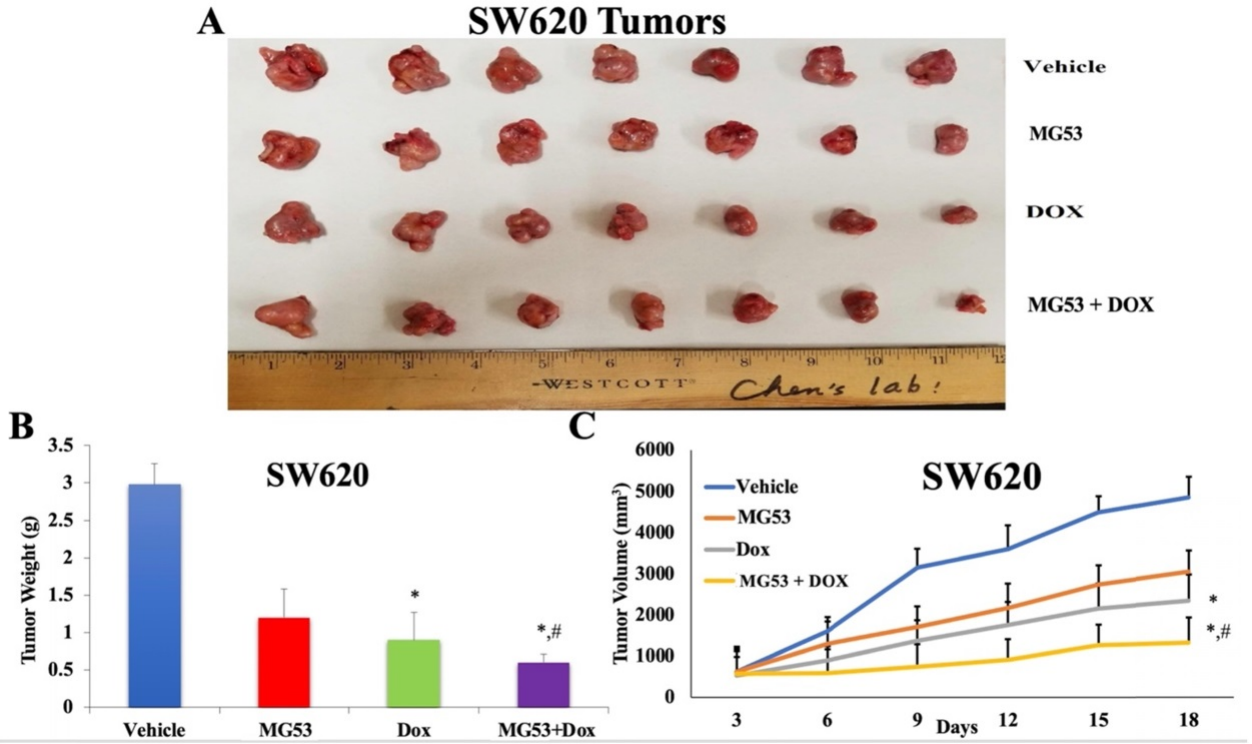
** Effect of rhMG53 on the volume and weight of tumors in SW620 tumor xenograft model.** (A) Images of SW620 tumors that were taken at the end of the treatment period. (B) The mean weight (n=7) of the SW620 tumors from the mice treated with vehicle, rhMG53, doxorubicin, and the combination of rhMG53 and doxorubicin, at the end of the 12-day treatment period. (C) The changes in tumor volume over time. Each point online graph represents the mean tumor volume (mm^3^) on each particular day after implantation. Error bars represent SD. *: p < 0.05 versus vehicle group; #: p < 0.05 versus the MG53 alone group.

**Figure 5:**
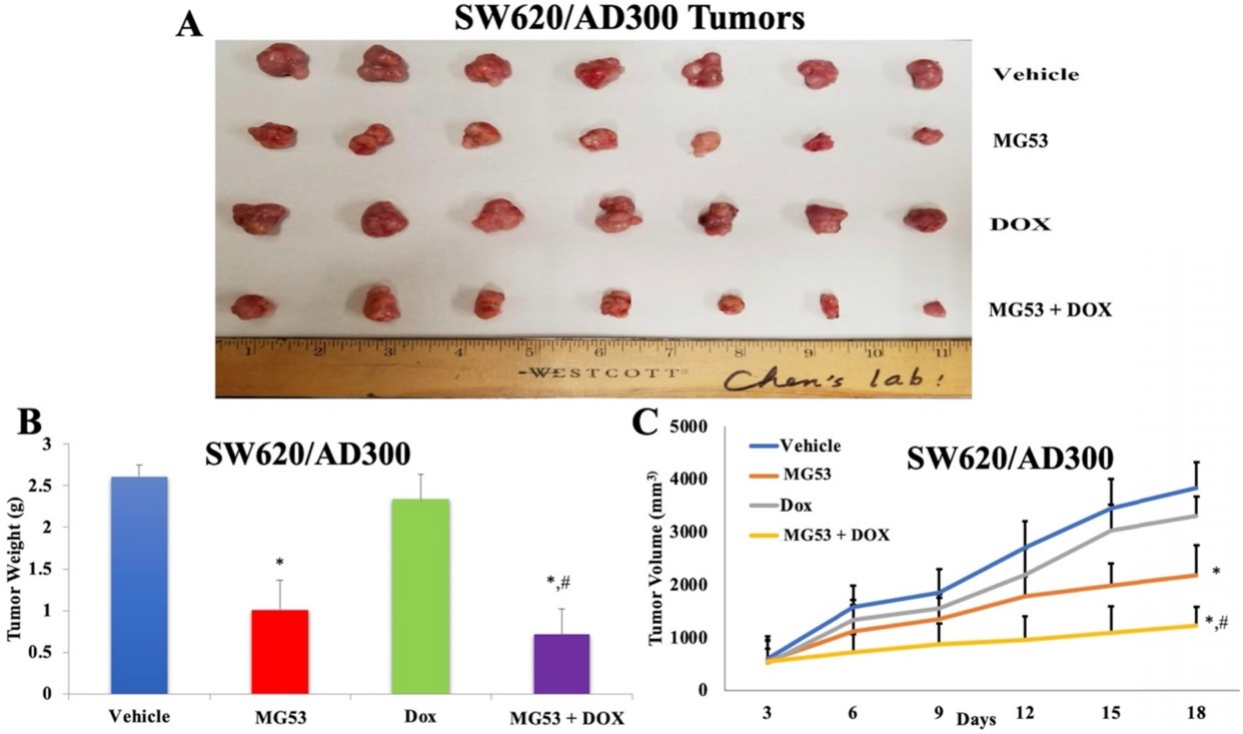
** Effect of rhMG53 on the volume and weight of tumors in SW620/AD300 tumor xenograft model.** (A) Images of SW620/AD300 tumors that were taken at the end of the treatment period. (B)The mean weight (n=7) of the SW620/AD300 tumors from the mice treated with vehicle, rhMG53, doxorubicin, and the combination of rhMG53 and doxorubicin, at the end of the 18-day treatment period. (C) The changes in tumor volume over time. Each point online graph represents the mean tumor volume (mm^3^) on each particular day after implantation. Error bars represent SD. *: p < 0.05 versus vehicle group; #: p < 0.05 versus the MG53 alone group.

Based on our promising *in-vitro* findings, we extended our study to evaluate the effect of rhMG53 in mouse tumor xenograft models of colorectal adenocarcinoma. The sensitive and drug resistant cell lines were inoculated into the flank region of mice to generate the parental and ABCB1 overexpressing tumor xenograft model, respectively. The animals were randomly divided into four groups (n=7) and treated as mentioned in 'Material and Methods'. As shown in Figures 4 and 5, the control saline group exhibited a significant increase in volume of tumors derived from transplanted SW620 (Figure 4A and 4C) and SW620/AD300 (Figure 5A and 5C) cells. Upon treatment with rhMG53 (2 mg/kg, q3d × 4, i.v.) or doxorubicin (2 mg/kg, q3d × 4, i.p.), tumor volume reduced significantly in the SW620 tumors. However, since the SW620/AD300 tumors are resistant to doxorubicin, treatment with doxorubicin alone (2 mg/kg, q3d × 4, i.p.) did not reduce the tumor growth or volume in the SW620/AD300 tumors. Interestingly, treatment with rhMG53 (2 mg/kg, q3d × 4, i.v.) significantly reduced the tumor growth and volume in SW620/AD300 tumors. Moreover, the combination of rhMG53 and doxorubicin further reduced the tumor growth and volume in both sensitive and drug resistant tumors. On day 18 of the treatment, we measured the tumor weights from every mouse. As shown in Figure 4B and 5B, a significant reduction in the tumor weight was observed in the rhMG53 and doxorubicin groups as compared to the vehicle control group.

### Assessment of potential toxicity of rhMG53

**Figure 6:**
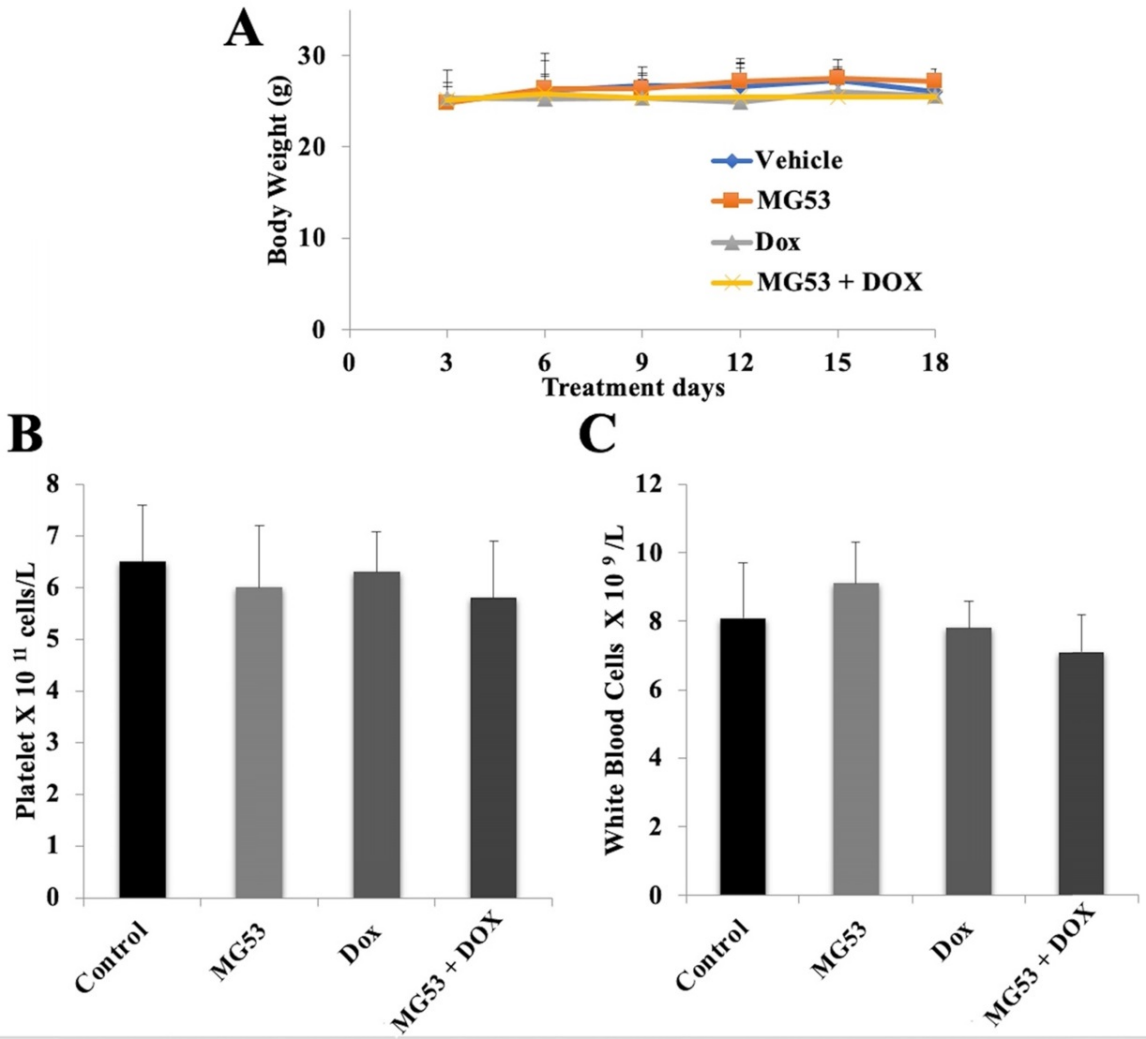
** Assessment of potential toxicity of rhMG53.** The body weight of animals bearing the sensitive and drug-resistant tumors is plotted over the 18-day treatment period (A). (B) Platelet and (C) white blood cell count were measured on day 18 of the treatment. Bar graphs represent the mean of the blood cell count, and error bars represent SD.

Systemic toxicity is one of the major concerns for any anti-cancer treatment. In order to evaluate the potential toxicity of rhMG53 alone or in combination with doxorubicin, we recorded the body weights of each animal every third day. Our results showed that the body weights of the animals did not change in any of the treatment groups. (Figure 6A). Furthermore, to assess any hematological toxicity, we did the blood cell count on day 18 of the study. As shown in Figures 6B and 6C, no significant change in the platelet and white blood cell count was observed in any of the treatment groups. Our findings show that rhMG53 when combined with doxorubicin exerts an anti-cancer effect on both parental and ABCB1 overexpressing tumors without any apparent weight loss or hematological toxicity.

## Discussion

Our findings, for the first time, show the effects of rhMG53 on colorectal carcinoma. We used cell proliferation assay to assess the effect of rhMG53 on both parental SW620 and doxorubicin-resistant SW620/AD300 cells. We found that rhMG53 did not impact the sensitivity of SW620/AD300 cells to doxorubicin treatment, yet it can inhibit the growth of the colorectal cancer cells. As shown in Table 1, doxorubicin alone exhibited higher IC_50_ in SW620/AD300 cells as compared to SW620 cells. Our results show that no significant changes in IC_50_ of SW620/AD300 cells to doxorubicin were observed with incubation of rhMG53, suggesting that rhMG53 did not directly impact ABCB1-mediated MDR. Using biochemical assay, we further found that rhMG53 treatment did not alter the protein expression of ABCB1 in SW620/AD300 cells.

Our present study demonstrated that rhMG53 has anti-proliferative effect on colorectal cancer cells regardless of their resistance to chemotherapeutic drugs. In our early study, we found that MG53 suppresses tumor progression and stress granule formation by modulating G3BP2 activity in non-small cell lung cancer 48. In the present study, we provide live cell imaging data to show that the exogenous rhMG53 protein can be taken up by the SW60 and SW60/AD300 cells (Figure 3), further supporting the intracellular action of rhMG53. In separate studies, we demonstrated that the exogenously applied rhMG53 can efficiently target the tumor cells 48. More studies are required to establish the connection between the mechanistic action of the intracellular MG53 on tumor cell growth.

The promising findings from our *in-vitro* studies inspired us to investigate the effect of rhMG53 in combination with doxorubicin in the parental and ABC overexpressing cancer cells using mouse tumor xenograft models 11, 49. While the xenograft formed by the implantation of the SW620/AD300 cells are resistant to doxorubicin treatment, the combination of rhMG53 and doxorubicin could inhibit their tumor growth and progression. rhMG53 in combination with doxorubicin did not produce significant hematological toxicity or weight loss to the animals during the study period, suggesting the safe nature of rhMG53 in the mouse model of colorectal carcinoma. However, the safety profile of the combination of rhMG53 and doxorubicin is yet to be established in large animal models and humans in future studies.

## Conclusions

In conclusion, our results show that rhMG53 inhibits proliferation of both drug-sensitive SW620 and doxorubicin resistant ABCB1 overexpressing SW620/AD300 cells, without altering the expression of ABCB1 protein. As evidenced by our *in-vivo* results, the combination of rhMG53 and doxorubicin attenuates the growth and volume of parental and ABCB1 overexpressing tumors. The combination of rhMG53 and doxorubicin presents a potential new regimen to treat cancer patients that are both susceptible and resistant to chemotherapy due to ABCB1.

## Supplementary Material

Supplementary figure and table.

## Figures and Tables

**Table 1:** The effect of rhMG53 on reversal of ABCB1-mediated MDR.

Treatment	IC_50_ ± SD^a^ (μM) (FR^b^)	
	SW620	SW620/AD300
Doxorubicin	0.18 ± 0.04 (1)	56.78 ± 2.35 (315.45)
+1 μM rhMG53	0.18 ± 0.06 (1)	55.14 ± 2.16 (306.34)
+3 μM rhMG53	0.17 ± 0.09 (0.9)	51.09 ± 3.01 (283.84)
+3 μM Verapamil	0.24 ± 0.09 (1.34)	1.22 ± 0.21 (6.78) ^c^

^a^ IC_50_ is presented as mean ± SD value of three independent experiments (n=3) each performed in triplicates ^b^ FR: Resistance fold is the IC_50_ value of substrate drug with or without inhibitor over the IC_50_ of substrate drug in parental cells without inhibitor ^c^ P < 0.05 compared to the control group in the absence of reversal agent

## Abbreviations

MDR: multidrug resistance
ABCB1: ATP-binding cassette subfamily B member 1
USFDA: US Food and Drug Administration
ABC: ATP-binding cassette
Pgp: P-glycoprotein 1
FBS: fetal bovine serum
DMSO: dimethyl sulfoxie
CO2: carbon dioxide
BSA: bovine serum albumin
WBC: white blood cells
