# Supplementary material for: MG53 inhibits cellular proliferation and tumor progression in colorectal carcinoma
Source: Int J Biol Sci. 2022 Aug 15;18(14):5221–9. doi: 10.7150/ijbs.67869 (PMC9461659; doi:10.7150/ijbs.67869)
Supplement: Supplementary file 1 — Supplementary figure and table. [file ijbsv18p5221s1.pdf]

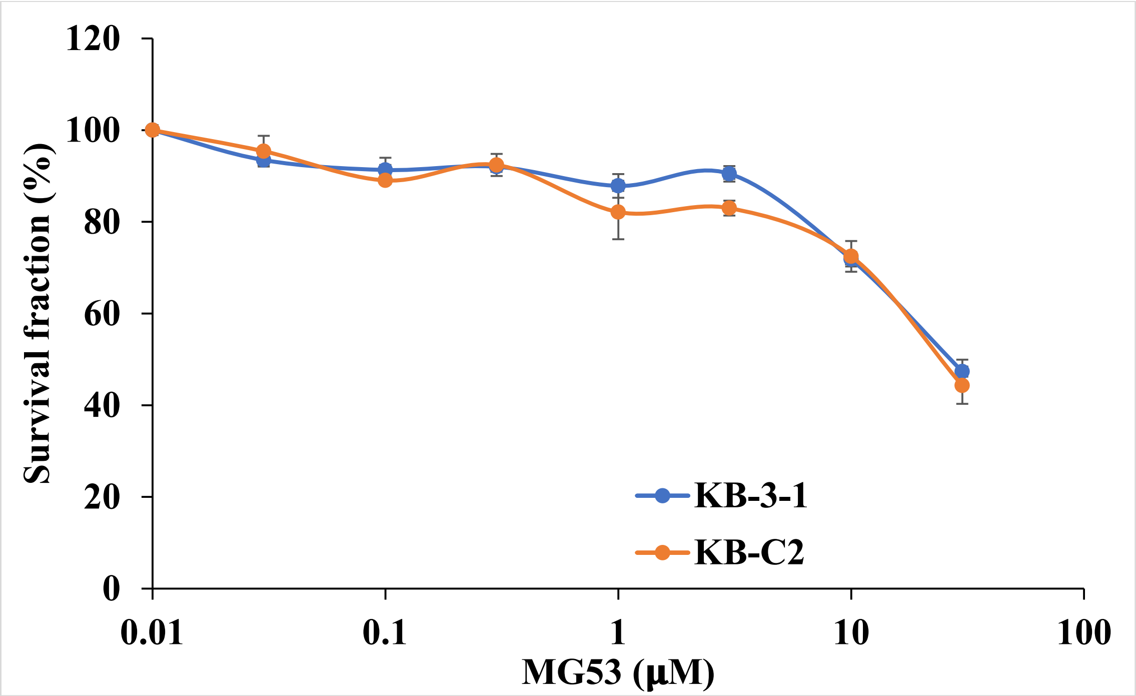

Figure S1: Anti-proliferative effects of rhMG53 on parental KB-3-1 and ABCB1-overexpressing KB-C2 cells. Cytotoxicity assay (MTT assay) was performed to evaluate the anti-proliferative effects of rhMG53 on parental and ABCB1-overexpressing cells. The data are representation of mean ± SD for three independent experiments performed in triplicates.

Table S1.

The effect of rhMG53 on reversal of ABCB1-mediated MDR

Treatment	$IC_{50} \pm SD$ [a] (µM) (RF [b])	
	KB-3-1	KB-C2
Doxorubicin	0.14 ± 0.01 (1.00)	7.20 ± 0.31 (51.47)
+ 0.3 µM rhMG53	0.12 ± 0.01 (0.84)	7.19 ± 0.26 (51.37)
+ 1 µM rhMG53	0.11 ± 0.01 (0.78)	6.56 ± 0.98 (46.85)
+ 3 µM rhMG53	0.11 ± 0.03 (0.80)	4.94 ± 1.06 (35.26)
+ 3 µM Verapamil	0.17 ± 0.01 (1.21)	0.56 ± 0.01 (3.97) [c]

[a] IC_{50} is presented as mean ± SD value of three independent experiments (n=3) each performed in triplicates

[b] RF: Resistance fold is the IC_{50} value of substrate drug with or without inhibitor over the IC_{50} of substrate drug in parental cells without inhibitor

[c] $P < 0.05$ compared to the control group in the absence of reversal agent
